# Linking Biocrust Architecture and Dispersal: Reproductive Ecology of Lichens of the Grit Crust in the Coastal Atacama Desert

**DOI:** 10.1111/1758-2229.70384

**Published:** 2026-07-05

**Authors:** Lina Werner, Janina Koziol, Patrick Jung

**Affiliations:** ^1^ XCEL—Extreme Cryptogam Ecology Lab University of Applied Sciences Kaiserslautern Kaiserslautern Germany; ^2^ Microbial Architecture University of Applied Sciences Kaiserslautern Kaiserslautern Germany; ^3^ WWHK—Department of Materials Science & Materials Testing, Institute QM3 University of Applied Sciences Kaiserslautern Kaiserslautern Germany; ^4^ Faculty of Natural Sciences and Technology Saarland University Saarbrücken Germany

**Keywords:** Caliciaceae, chlorolichens, grit crust, spores, *Trebouxia*

## Abstract

Biological soil crusts are essential components of arid ecosystems, yet the establishment and dispersal of lichen‐dominated crusts remain poorly understood. In the coastal Atacama Desert, a unique biocrust type—the grit crust—is formed by minute chlorolichens colonising mobile quartz particles. We investigated the developmental stages and dispersal mechanisms of these micro‐lichens using high‐resolution digital microscopy, scanning electron microscopy and micro‐manipulative direct PCR. Microscopic structures on individual quartz grains included melanised fungal micro‐colonies, exploratory hyphal networks, free‐living green algal colonies and developing lichen thalli. Molecular analyses identified mycobionts of the Caliciaceae alongside photobionts of the green algal genus Trebouxia, occurring in both lichenised and non‐lichenised states. The presence of free‐living algal cells on grit surfaces suggests that unassociated photobionts function as environmental reservoirs priming symbiosis formation. Our findings support a sequential assembly model in which airborne fungal spores colonise mineral substrates before encountering compatible photobionts. Multiple dispersal vectors—including fungal spores, lichen fragments and algal cells carried by wind and dust—confer high colonisation and recovery potential to grit crust communities, likely driving the rapid turnover, spatial heterogeneity and productivity characteristic of this exceptional lichen‐dominated desert biocrust.

## Graphical abstract

Microscopic analysis of Atacama Desert grit crust revealed a sequential assembly process in which airborne fungal spores of the Caliciaceae colonise quartz particles before encountering compatible *Trebouxia* photobionts. Free‐living algal cells on grit surfaces function as environmental reservoirs for symbiosis initiation, while multiple wind‐driven dispersal vectors collectively sustain the colonisation and recovery potential of these micro‐lichen communities.

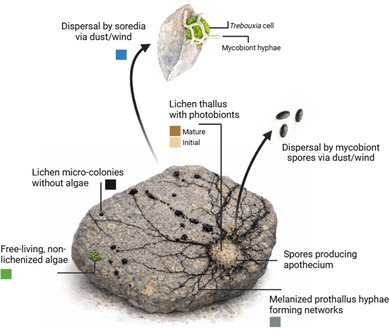

## 1 Introduction

Biological soil crusts (biocrusts) are complex microbial assemblages inhabiting the uppermost millimetres of soil surfaces in arid and semi‐arid ecosystems worldwide. These communities consist of cyanobacteria, algae, lichens, fungi, bryophytes and associated heterotrophic microorganisms, which collectively stabilise soil surfaces, influence hydrological processes and contribute significantly to carbon and nitrogen cycling in drylands (Belnap and Lange 2001; Bowker et al. 2018; Weber et al. 2022). In (hyper)arid deserts and drylands, where vascular plant cover is extremely sparse, biocrust organisms often represent the dominant form of biological activity and play an essential role in ecosystem functioning (Pointing and Belnap 2012).

The Atacama Desert in northern Chile is widely regarded as one of the driest environments on Earth where biocrusts have been detected (Wang et al. 2017; Fernandez‐Murillo et al. 2026). Despite annual precipitation levels often below 2 mm, the coastal range of the desert supports surprisingly diverse microbial communities due to the frequent occurrence of advective fog events (locally termed camanchaca) originating from the Pacific Ocean (Lehnert et al. 2018; García et al. 2021). These fog inputs create localised moisture gradients that strongly influence microbial colonisation and productivity. Within this system, several unique forms of biocrust have been described, including the recently recognised ‘grit crust’, a distinctive microbial community dominated by lichens growing on small quartz and granitoid particles typically measuring 2–6 mm in diameter (Jung, Baumann, Lehnert, et al. 2020; Jung et al. 2025). The grit crust is best understood not as a discrete biocrust type but as an ecological intermediate between a saxicolous lichen community and a fully developed biological soil crust (Weber et al. 2022; Jung et al. 2020). This interpretation rests on the substrate behaviour of its dominant lichens: the core Caliciaceae are typically epilithic or epiphytic taxa that here colonise the surfaces of granitoid pebbles, whereas co‐occurring terricolous lichens such as *Acarospora conafii* and an undescribed *Wetmoreana*‐related species grow mainly in the fine substrate between the grits and are never fully developed on the stones themselves (Jung, Brand, et al. 2024). The community therefore spans a continuum from organisms still bound to a rock surface to organisms that have already committed to a soil habitat, capturing the very transition by which biocrusts are thought to emerge from rock‐dwelling pioneer assemblages. Functionally, this transitional position is reinforced by the *Trebouxia‐*driven biomass accumulation (Jung, Brand, et al. 2024), bio‐weathering and carbon fixation of the grit crust, processes that progressively convert the pebble matrix into finer mineral substrate (Jung, Baumann, Emrich, et al. 2020) and thereby drive the system away from its saxicolous origin and towards a true soil crust.

The grit crust is dominated by small chlorolichens belonging primarily to members of the Caliciaceae and related taxa with diverse lineages of *Trebouxia* spp. as green algal photobionts (Jung, Brand, et al. 2024). These micro‐lichens form extremely small thalli that frequently cover only a fraction of the surface of individual grit stones. Despite their minute size, the lichens occur in high densities and exhibit rapid turnover across the substrate surface, producing a dynamic mosaic of developmental stages ranging from early colonisation structures to mature reproductive thalli. The spatial organisation of these lichens is strongly influenced by the availability of suitable microhabitats on the stone surface, as well as by dispersal processes that introduce fungal spores and photobiont cells into the system (Jung, Brand, et al. 2024).

Lichens are symbiotic associations typically composed of a filamentous fungal partner (the mycobiont) and one or more photosynthetic partners (photobionts), most commonly green algae of the genus *Trebouxia* or cyanobacteria (Honegger 2009). For a long time, lichens were interpreted as tightly integrated dual symbioses. However, recent studies have revealed that lichen symbioses are far more complex and involve diverse bacterial communities, accessory fungi, and dynamic metabolic exchanges between partners (Spribille et al. 2016; Smith et al. 2020). Furthermore, increasing evidence suggests that many photobionts, particularly species of *Trebouxia*, are not restricted to the lichen symbiosis but can also occur as free‐living algae in environmental reservoirs such as soil, bark, or rock surfaces (Ahmadjian 1988; Veselá et al. 2024). These free‐living photobiont populations are thought to represent an important source of symbiotic partners during lichen establishment and dispersal but have rarely been documented (Veselá et al. 2024).

Lichen reproduction and dispersal strategies linked to certain environmental factors such as water availability, wind and/or dust strongly influence colonisation patterns in extreme environments. Lichens can disperse either via vegetative propagules containing both partners (e.g., soredia or isidia) or via fungal spores that must subsequently re‐associate with compatible photobionts (Honegger 2009). In many crustose lichens, sexual reproduction via ascospores produced in apothecia is the dominant dispersal mode. After dispersal, fungal spores germinate and form exploratory hyphae that search for compatible photobiont cells in the surrounding environment (Sanders 2014). Experimental studies have shown that successful lichenisation may involve multiple intermediate stages, including temporary free‐living phases of both symbiotic partners (Pichler et al. 2023). Recent conceptual frameworks describe lichen assembly as a dynamic ecological process involving environmental reservoirs of symbiotic partners, spatial dispersal and metabolic signalling between fungi and algae (Armaleo et al. 2019; Pichler et al. 2023).

In deserts, the assembly of lichen symbioses may be particularly challenging due to the extremely limited availability of water and nutrients, influencing the actual metabolic activity phase of the organisms. Nevertheless, lichens are among the most conspicuous biological components of desert rock surfaces and desert pavements. Studies from the Atacama Desert have demonstrated that lichenised fungi frequently colonise lithic substrates in association with specialised photobiont lineages that show strong and various adaptations to extreme radiation and desiccation (Vondrak and Kubásek 2013; Beckett et al. 2021). Moreover, several studies have suggested that free‐living photobionts may persist independently in desert soils and rock surfaces, providing a potential pool of symbiotic partners for newly germinating fungal spores (Veselá et al. 2024). Such environmental reservoirs of photobionts may play a crucial role in shaping the assembly dynamics of desert lichen communities.

Although dispersal and colonisation processes have been studied in several lichen systems, the developmental stages, detailed structures and functions of micro‐lichens as part of biocrusts in natural environments remain poorly documented. In the grit crust of the Atacama Desert, microscopic observations have revealed the presence of small rounded black micro‐colonies, venous networks of fungal prothallus hyphae extending across quartz surfaces, and isolated green algal colonies occurring in close proximity to developing lichen thalli (Jung, Baumann, Lehnert, et al. 2020; Jung et al. 2025). These structures likely represent different stages of lichen establishment, including early fungal growth phases, photobiont colonisation events and the formation of initial lichen thalli, all of which have different functions such as involvement in bio‐weathering activities (Jung, Baumann, Emrich, et al. 2020).

Recent methodological advances allow these early colonisation processes to be investigated directly. Micro‐manipulative sampling combined with direct PCR, for example, enables the genetic identification of microscopic structures such as isolated fungal micro‐colonies, prothallus networks, early lichen thalli and free‐living algal colonies (Jung, Briegel‐Williams, et al. 2024; Jung et al. 2025). Linking these structures to their fungal and photobiont partners provides a powerful approach to reconstruct the sequence of events underlying lichen establishment on individual substrate particles and to test hypotheses about symbiosis assembly in situ.

Based on these observations, we hypothesise that caliciacean lichen colonisation including trebouxioid photobiont lineages of grit stones in the Atacama Desert occurs through a sequential assembly in which different pools of the symbiotic partners, dispersal strategies, and functional structures are involved. From this conceptual framework, two specific hypotheses arise: (i) lichenised structures are dispersed through airborne dust and wind transport and (ii) free‐living, non‐lichenised photobionts, particularly *Trebouxia*, occur independently on grit stones and serve as environmental reservoirs that facilitate the formation of lichen symbioses during early colonisation stages.

To test this, we used a combination of digital microscopy, scanning electron microscopy and micro‐manipulative direct PCR and we analysed the occurrence and spatial relationships of fungal micro‐colonies, prothallus networks, early lichen thalli and mature reproductive structures on individual quartz grit stones. In addition, we assessed the occurrence of free‐living green algal photobionts and evaluated their potential role as environmental reservoirs for lichen assembly. By integrating microscopic observations with molecular identification of fungal and algal partners, this study aims to elucidate the mechanisms underlying lichen dispersal and symbiosis formation in one of the most extreme terrestrial ecosystems on Earth.

## 2 Methods

### 2.1 Site Description and Sampling

**FIGURE 1:**
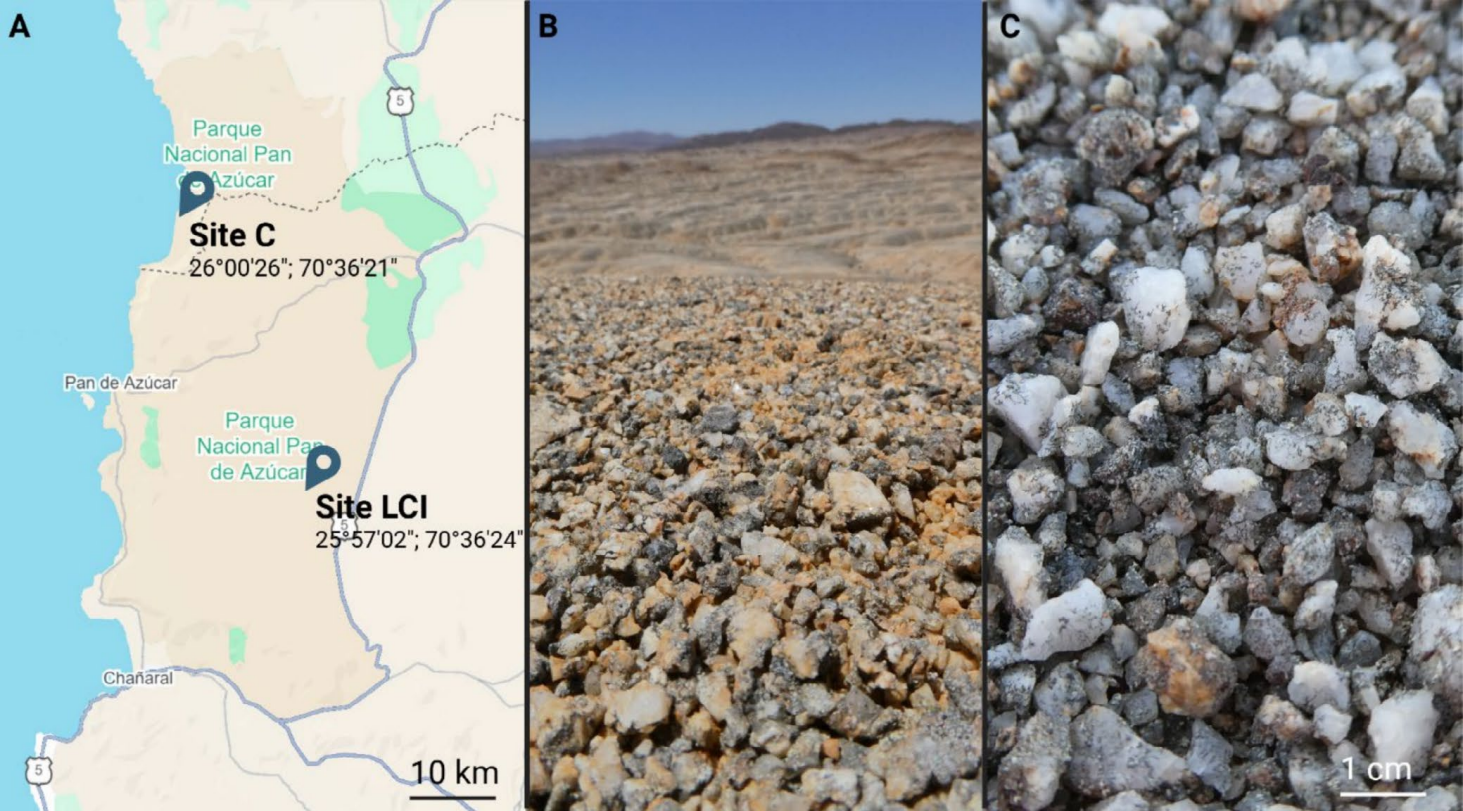
Sampling site. (A) Map of the National Park and the two sampling sites C and LCI, (B) landscape photography showing the grit crust, (C) close‐up of colonised grit stones.

The study was conducted in Pan de Azúcar National Park (Figure 1A), located in the coastal range of the Atacama Desert in northern Chile. The park covers approximately 440 km^2^ and represents a characteristic hyperarid coastal desert ecosystem influenced by the interaction of Pacific Ocean fog and extremely low precipitation (Lehnert et al. 2018; Jung et al. 2025).

The landscape is characterised by sparsely vegetated desert terrain composed of granitoid substrates and coarse soil particles. In many areas, the soil surface is dominated by a distinctive biological soil crust type known as grit crust (Figure 1B,C), in which lichens colonise small granitoid pebbles of approximately 2–6 mm diameter, forming blackish patch‐like patterns visible across the ground surface (Jung, Baumann, Lehnert, et al. 2020).

The two sampling sites within the park represent two contrasting sites, which are characterised by water availability and microbial colonisation patterns. Among the investigated locations, site C is situated close to the coastal ridge (approximately 100 m distance) and is frequently exposed to fog events (Jung et al. 2025). As a result, this site represents one of the most humid environments within the study area, showing relatively dense microbial colonisation and higher vegetation cover including cacti and *Euphorbia* shrubs. In contrast, site LCI represents an intermediate location within the park, characterised by a flat landscape without vascular vegetation and a moderate influence of coastal fog, resulting in intermediate coverage of the grit crust (Jung et al. 2025).

Samples of the biocrust were taken by collecting 250 g of grit crust cover from two sites using a broad brush. The material was air‐dried and stored in the dark.

Dust was collected as described in Jung, Baumann, Emrich, et al. (2020) from a plastic sheet (31 × 43 cm), which had been carefully attached to the ground by pegs. After 10 days of exposure to the open air, the dust was wiped off with a sterile filter cotton mesh and placed in a plastic tube for transport.

### 2.2 Cross‐Sections, Light Microscopy and Micro‐Colony Area Calculation

To quantify single, black micro‐colonies of lichens on grit stones, digital microscopy images were acquired using a VHX‐7000 digital microscope (Keyence Corporation, Osaka, Japan). A total of 100 individual grit stones per sampling location were imaged under identical illumination and magnification settings to ensure comparability among samples. Images of single grit stones were taken to document the complete surface of each stone.

Image analyses were performed using the integrated VHX analysis software (Keyence). The software's classification function was trained to distinguish between (i) the light‐coloured quartz substrate, (ii) lichen thalli, (iii) the venous prothallus networks and (iv) single, round and black micro‐colonies as early developmental stages in lichen formation visible on the stone surfaces. Training of the segmentation algorithm was conducted by manually selecting representative regions corresponding to the four above mentioned categories. Following classifier training, automated binary segmentation (black–white thresholding) was applied to all images. In the resulting masks, white pixels represented uncolonised stone surfaces, lichen thalli or pro‐thalli, whereas black pixels represented areas covered by lichen micro‐colonies. The relative surface coverage (%) of micro‐colony structures was calculated as the proportion of black pixels relative to the total image area for each stone. All images were processed using identical segmentation parameters to ensure consistency across samples from the two study locations. Differences in micro‐colony surface coverage between the two sampling locations were tested using a Mann–Whitney U test, as the data did not meet the assumptions of normality. Statistical significance was assessed at *α* = 0.05, and all analyses were performed using SigmaPlot (Systat Software Inc., San Jose, CA, USA).

Lichen squamules were carefully removed from a single grit in a hydrated state with a sterile needle and transferred to a freezing microtome. Thin sections of 25 μm thickness were prepared and transferred to a drop of water on an object slide and visualised using a light microscope (Axioskop, Zeiss, Germany with DIC optics) under 630 magnification and oil immersion.

Various colonised grit stones were embedded in water‐free resin and cut into blocks after drying and hardening. The blocks were carefully ground down to a thickness of 30 μm and mounted between a glass objective slide and a cover slide. These thin sections were visualised with an epifluorescence microscope (Axioskop; HBO 50; Zeiss) as described above by exciting the autofluorescence of the mycobiont and the photobionts (blue–violet 395–440 nm excitatory filter; 460 nm chromatic beam splitter; 470 nm barrier filter).

### 2.3 Scanning Electron Microscopy

Scanning electron microscopy (SEM) and energy‐dispersive X‐ray spectroscopy (EDS) analyses were performed to investigate the surface morphology and elemental composition of several single representative air‐dried grit stones. The measurements were carried out using a TESCAN Clara Mark III SEM (Tescan, Brno, Czech Republic) equipped with an Octane Elect EDS system (EDAX/AMETEK, Mahwah, NJ, USA), allowing high‐resolution imaging as well as elemental mapping. All investigation were conducted at an acceleration voltage of *U* = 10 keV, a beam current of approx. I≈3 nA, and a working distance of WD≈10 mm. Prior to analysis, the samples were sputter‐coated with a approx. 3 nm thick gold layer to render the surface conductive and to minimise charging effects using a Cressington sputter coater 108 (Cressington Scientific Instruments Ltd., Watford, UK) in combination with the thickness controller MTM‐20.

Secondary electron imaging was used to obtain detailed information on the surface topography of the lichen thallus structures, while EDX mappings were acquired to visualise the spatial distribution of the detected elements across the investigated areas.

### 2.4 Direct PCR Approach

Single dry grit stones covered with lichens were placed under a binocular (Stemi 508, Carl Zeiss Microscopy GmbH, Jena, Germany) to pick micro‐samples with sterile metal needles from (i) fully developed mature lichen thalli, (ii) small, initial thalli, (iii) black venous prothallus networks, (iv) black, single micro‐colonies, (v) green colonies. The amount of approximately one sand grain was removed from the lichen material and placed in PCR reaction tubes filled with lysis buffer and proteinase K of the Platinum Direct PCR Universal Master Mix kit (Thermo Fisher Scientific, Waltham, MA, USA) as described in Jung, Briegel‐Williams, et al. (2024). In addition to material from grit stones, collected dust was visualised under a binocular and single sand grains with lichen material attached were picked using sterile metal syringes and transferred to lysis buffer. For the lysis, the tubes were placed in a thermocycler (MiniAmp Thermal Cycler; Thermo Fisher Scientific, Waltham, MA, USA) for two minutes at 98°C.

For amplification of the ITS1 gene of the mycobiont the primers ITS1f (Gardes and Bruns 1993) and LR3 (Friedl and Rokitta 1997) were used, for the green algal photobionts the nuclear internal transcribed spacer of the ribosomal DNA (ITS, ITS1, 5.8S), ITS2 was amplified using the primers ITS1T and ITS4T (Kroken and Taylor 2000) with the identical lysate as DNA template. Success of amplification was checked by gel electrophoresis. PCR products that contained sufficient DNA concentration were cleaned using the NucleoSpin Gel and PCR Clean‐up Kit (Marchery Nagel, New England, Canada) and sent to Genewiz (Göttingen, Germany) for Sanger sequencing. Sequences were submitted to NCBI GenBank given the accession numbers in the phylogenetic trees.

### 2.5 Phylogenetic Reconstructions

The generated sequences were merged and the primers were trimmed using the software Geneious Prime (2022.01.1). Sequences for individual loci were assigned based on the NCBI GenBank dataset using the Basic Local Alignment Search Tool (BLAST) of Mega X (Kumar et al. 2018). Then, multiple sequence alignments were performed using the multiple sequence alignment method (Muscle) algorithm (Edgar 2004) implemented in Mega X. For each dataset the evolutionary model that was best suited to the database used was selected on the basis of the lowest Akaike Information Criterion (AIC) value and calculated in Mega X which was GTR + G + I for both. The phylogenetic reconstructions were calculated using NGPhylogeny.fr (Lemoine et al. 2019) with 500 bootstrap replications.

Besides Maximum Likelihood analyses (ML), Bayesian Inference (BI) was also calculated for each of these trees using Mr. Bayes 3.2.1 for the later analysis (Ronquist and Huelsenbeck 2003).

The tree topologies obtained by the BI method did not contradict the ML trees; thus, only the ML trees are shown.

## 3 Results

### 3.1 Identified Lichen Structures and Developmental Stages on Grit Stones

**FIGURE 2:**
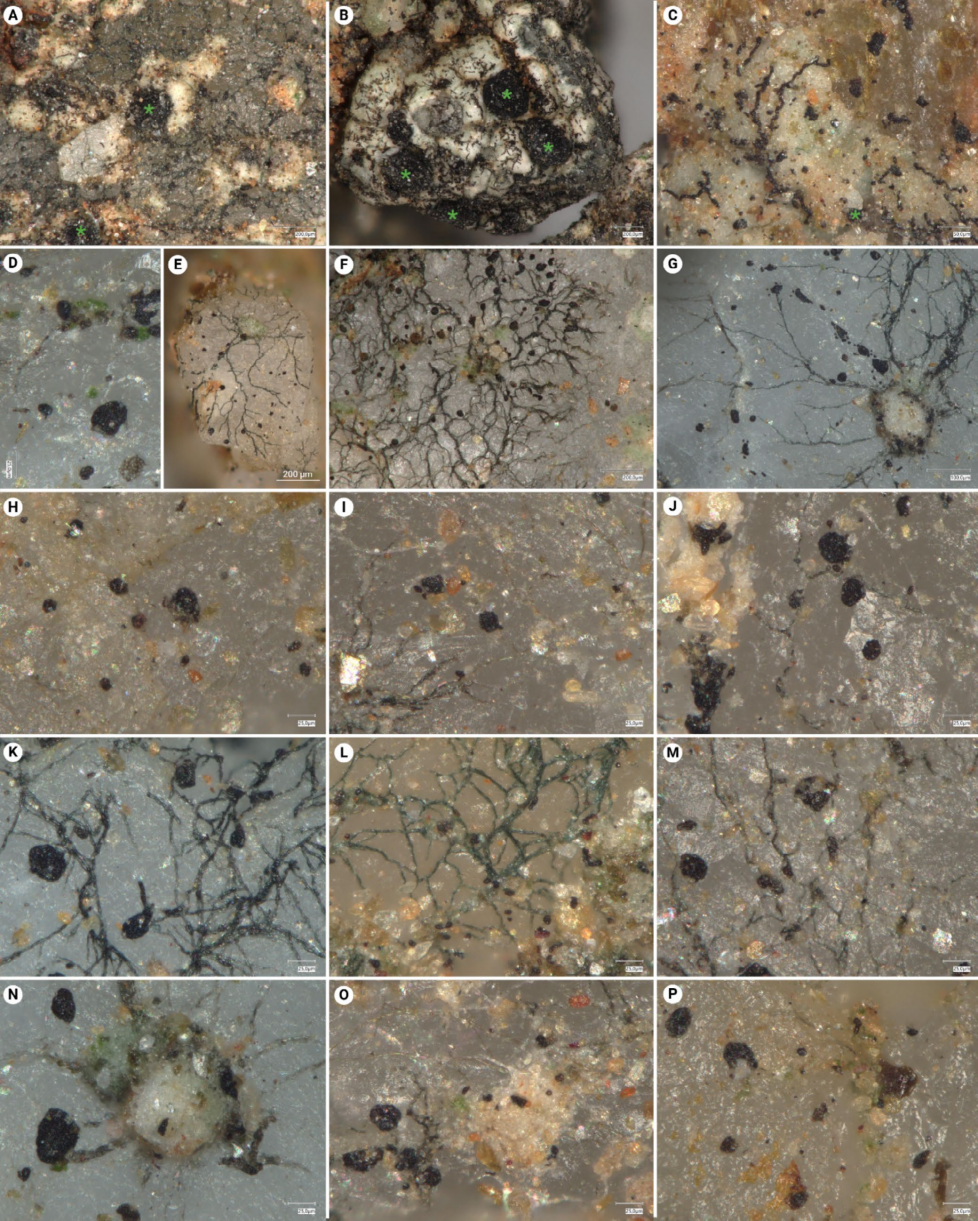
Lichen stages on grit stones. (A–C) mature lichen thalli with apothecia (asterisk). (D) round black micro‐colonies next to free‐living, non‐lichenised green algal colonies (E–G) mature lichen thalli with extended prothallus network and rounded, black micro‐colonies. (H–J) black micro‐colonies. (K–M) rounded, black micro‐colonies with prothallus networks without algae. (N–P) initial thalli showing early lichenisation of photobionts connected to black micro‐colonies via prothallus networks.

Microscopic examination of quartz grit stones revealed a wide range of lichen developmental stages occurring simultaneously on individual particles. Mature lichen thalli with well‐developed apothecia were frequently observed on the surface of grit stones (Figure 2A–C). These thalli typically formed compact, pale crustose structures that were attached to the quartz substrate.

In addition to mature thalli, numerous smaller structures were observed across the grit surfaces. Round, black micro‐colonies occurred frequently as isolated structures or in close spatial proximity to other lichen stages (Figure 2D,H–J). These colonies typically measured between approximately 20–100 μm in diameter and showed no visible association with photobiont cells under light microscopy.

Venous prothallus networks of dark fungal hyphae were also commonly observed extending across the quartz surfaces (Figure 2E–G,K–M). These networks formed branching structures connecting different colonisation points on the stone surface. In several cases, these prothallus networks were connected to round black micro‐colonies or small initial thalli. Some prothallus structures occurred independently without visible algal partners (Figure 2K–M).

Early stages of lichen formation were represented by small, pale thalli associated with surrounding hyphal networks (Figure 2N,P). These structures showed the first signs of lichenisation, with green photobiont cells visible within the developing thallus tissue. In several cases, these initial thalli were spatially connected to nearby black fungal micro‐colonies via prothallus hyphae.

**FIGURE 3:**
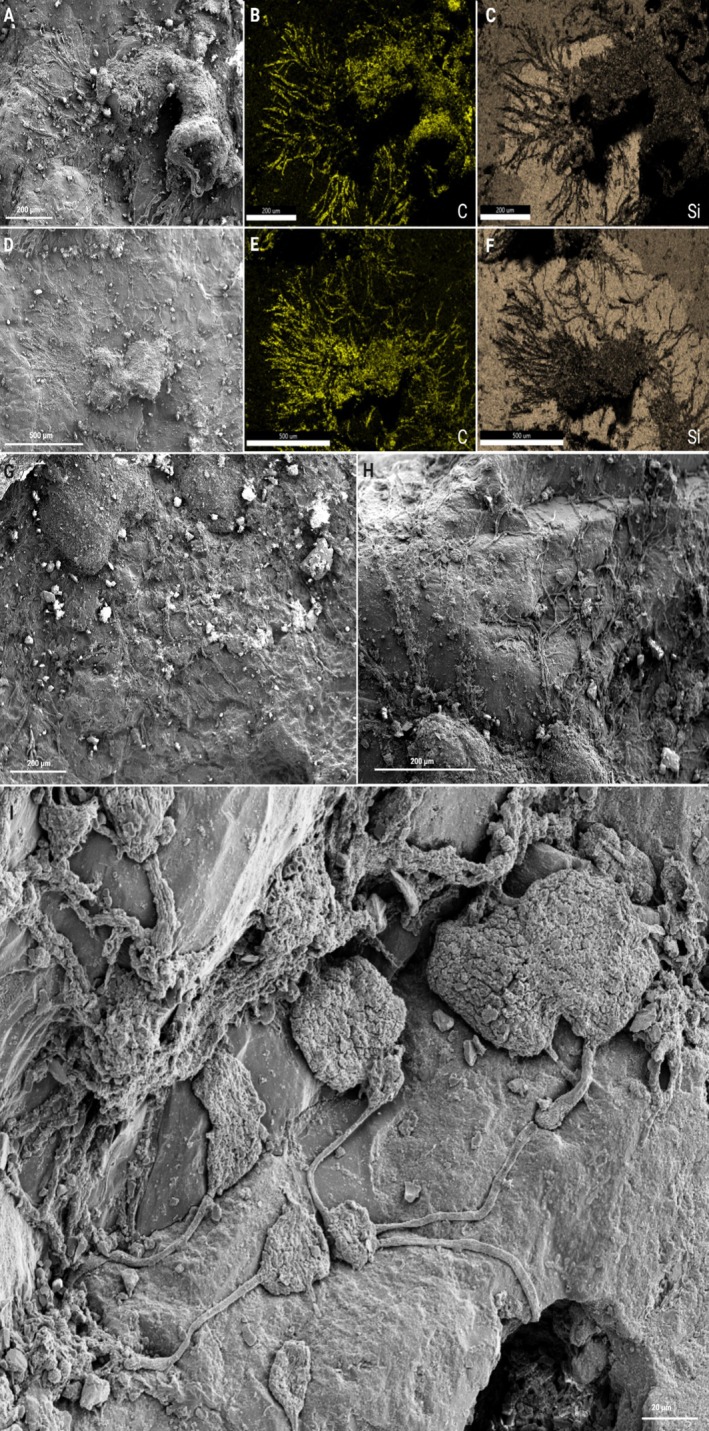
Scanning electron microscopy (SEM) and energy‐dispersive X‐ray spectroscopy (EDX) of lichen thallus structures. (A–F) lichen thallus and prothallus expansion showing carbon mapping in B, E and silica mapping in C, F. In the EDX element maps, signal intensity is proportional to the relative surface concentration of the mapped element, with brighter regions indicating higher abundance. The carbon maps (B, E) therefore highlight carbon‐rich organic material (fungal hyphae/algal cells of the thallus/prothallus) as the brighter signal, whereas carbon‐poor areas appear dark. Conversely, the silicon maps (C, F) show the silica‐rich quartz substrate as the brighter signal, with darker zones corresponding to areas where biological material overlies and masks the underlying mineral. The complementary distribution of carbon and silicon demonstrates the clear spatial separation between lichen biomass and the quartz grit. (G) lichen thallus in the left corner with prothallus hyphae extending over the quartz surface. (H) lichen thallus on the bottom with lichen prothallus network. (I) Micro‐colonies with scout hyphae.

Scanning electron microscopy (SEM) revealed detailed structural organisation of lichen thalli and associated prothallus networks on quartz grit surfaces (Figure 3A–I). Mature lichen thalli formed compact, three‐dimensional structures firmly attached to the mineral substrate (Figure 3A,D). Energy‐dispersive X‐ray (EDX) mapping showed a clear spatial separation between biological and mineral components, with carbon signals corresponding to fungal and algal biomass and silica signals associated with the quartz substrate (Figure 3B,C,E,F).

Prothallus hyphae extended radially from established thalli, forming interconnected networks across the quartz surface (Figure 3G,H). These hyphal structures closely followed microtopographic features of the mineral substrate and frequently bridged gaps between distinct colonisation sites. In several instances, prothallus networks connected mature thalli with adjacent micro‐colonies or extended into previously uncolonised areas.

High‐magnification images further revealed small, rounded micro‐colonies associated with thin exploratory (‘scout’) hyphae radiating outward into the surrounding substrate (Figure 3I). These structures were typically located at the periphery of established networks or in spatial isolation, indicating early stages of surface colonisation.

**FIGURE 4:**
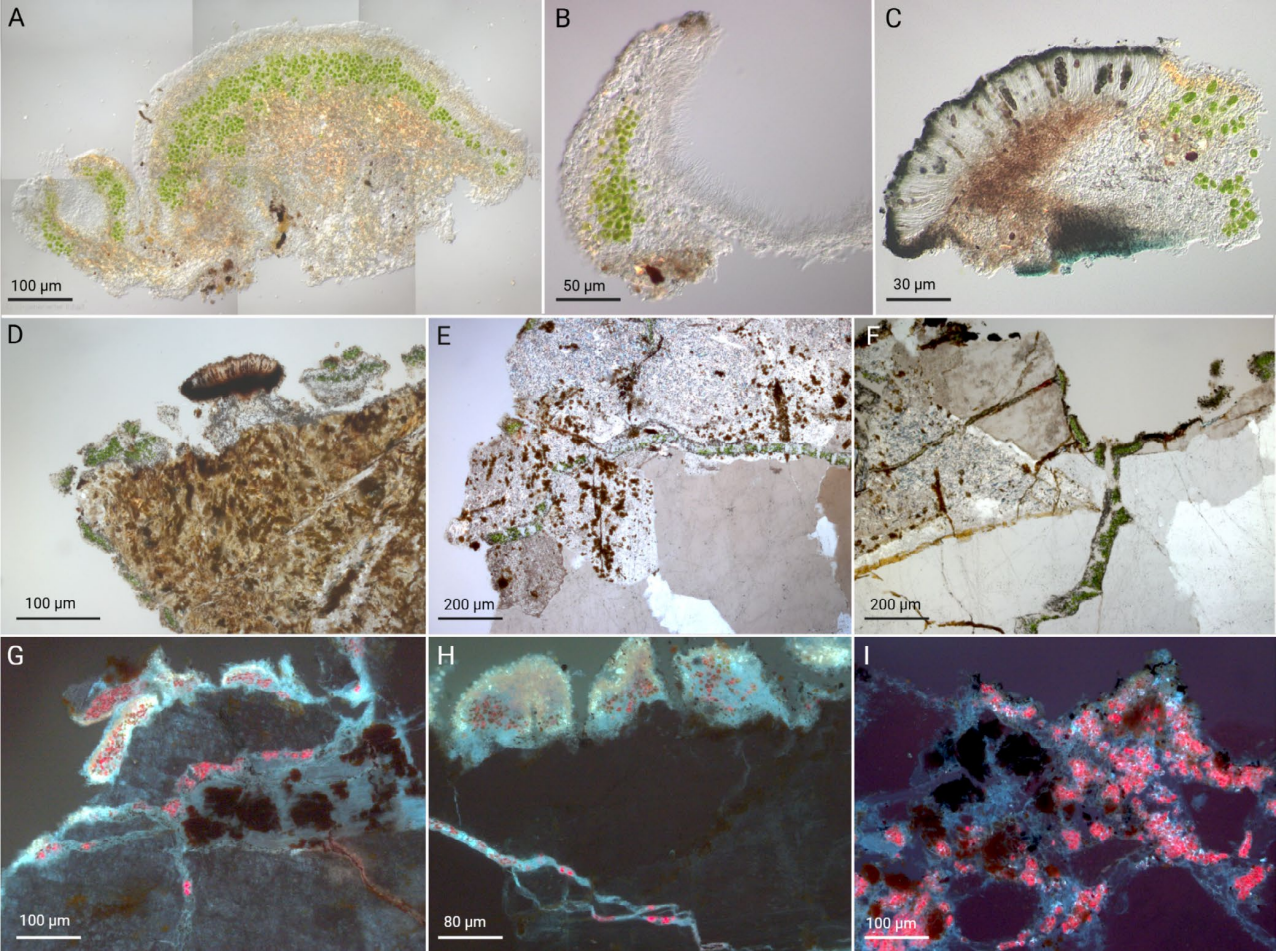
Lichen structures based on thin‐sections. (A) lichen thallus detached from grit stone with pigmented upper cortex, trebouxioid photobiont layer and medulla with mineral fragments. Note the pycnidium with spores on the left. (B) thin‐section of pycnidium with transparent spores. (C) thin‐section of apothecium with melanised spores. (D–F) thin‐section of resin embedded specimens showing lichen structures with fungal hyphae and algae in inner fissures of the grit stones. (G–I) autofluorescence of thin sections showing white fungal hyphae and red green algae in channels and deeper structures of the grit lithomatrix. Under blue–violet excitation (395–440 nm; see Methods), the two symbionts are distinguished by their autofluorescence: Fungal hyphae of the mycobiont emit in the whitish range and appear white, whereas the chlorophyll of the trebouxioid photobiont emits in the red and appears red. The red signal in deeper channels and fissures (G–I) thus marks the position of photobiont cells within the grit lithomatrix, while the white network traces the surrounding fungal hyphae.

Thallus thin sections showed a pigmented upper cortex of the lichen, a photobiont layer of trebouxioid algae and a medulla layer with mineral contact as well as (non melanised) spores in pycnidia and (melanised) in apothecia (Figure 4A–C). Cross‐sections of grit stones showed lichen structures including hyphae and algal cells penetrating into deeper areas of the stones such as along cracks and fissures forming extended channels (Figure 4D–I).

### 3.2 Colonisation Rate of Fungal Micro‐Colonies

**FIGURE 5:**
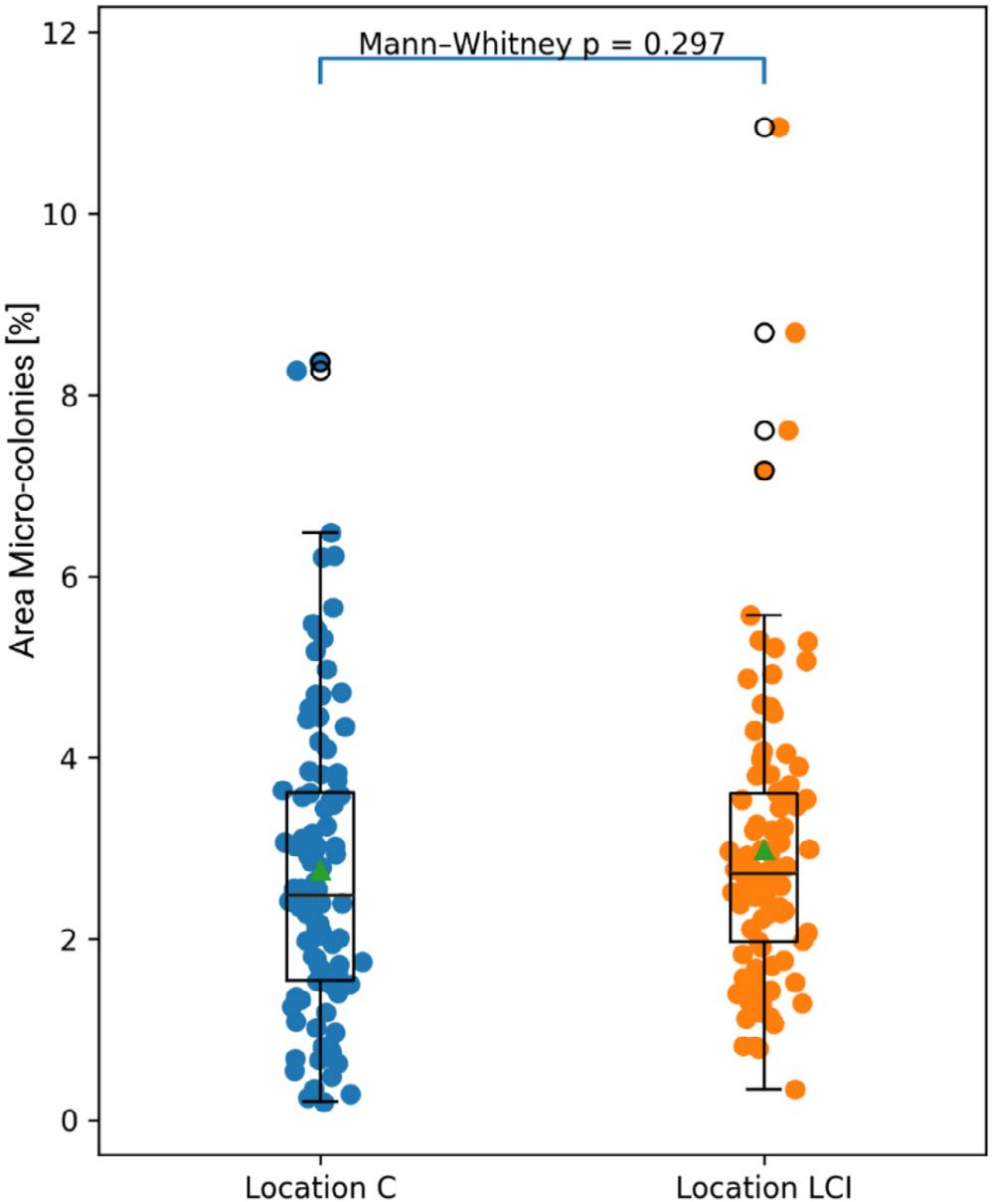
Boxplots with Jitter dots showing the colonisation rate of round, black lichen micro‐colonies per grit stone from two locations. Median location C = 2.49%, IQR = 1.54–3.62; median location LCI = 2.73%, IQR = 1.97–3.61; *n* = 100; Mann–Whitney *U* test, *U* = 4432.5, *p* = 0.297.

Quantitative image analysis of 100 grit stones per sampling location revealed that black fungal micro‐colonies covered only a small fraction of the total stone surface area. The median surface coverage of micro‐colonies was 2.49% at location C (IQR = 1.54–3.62) and 2.73% at location LCI (IQR = 1.97–3.61) (Figure 5). Statistical comparison using a Mann–Whitney U test revealed no significant difference in micro‐colony coverage between the two locations (*U* = 4432.5, *p* = 0.297). The distribution of values showed considerable variability among individual grit stones within both locations, with most stones exhibiting only small areas covered by micro‐colonies.

### 3.3 Molecular Identification of Fungal and Algal Partners

**FIGURE 6:**
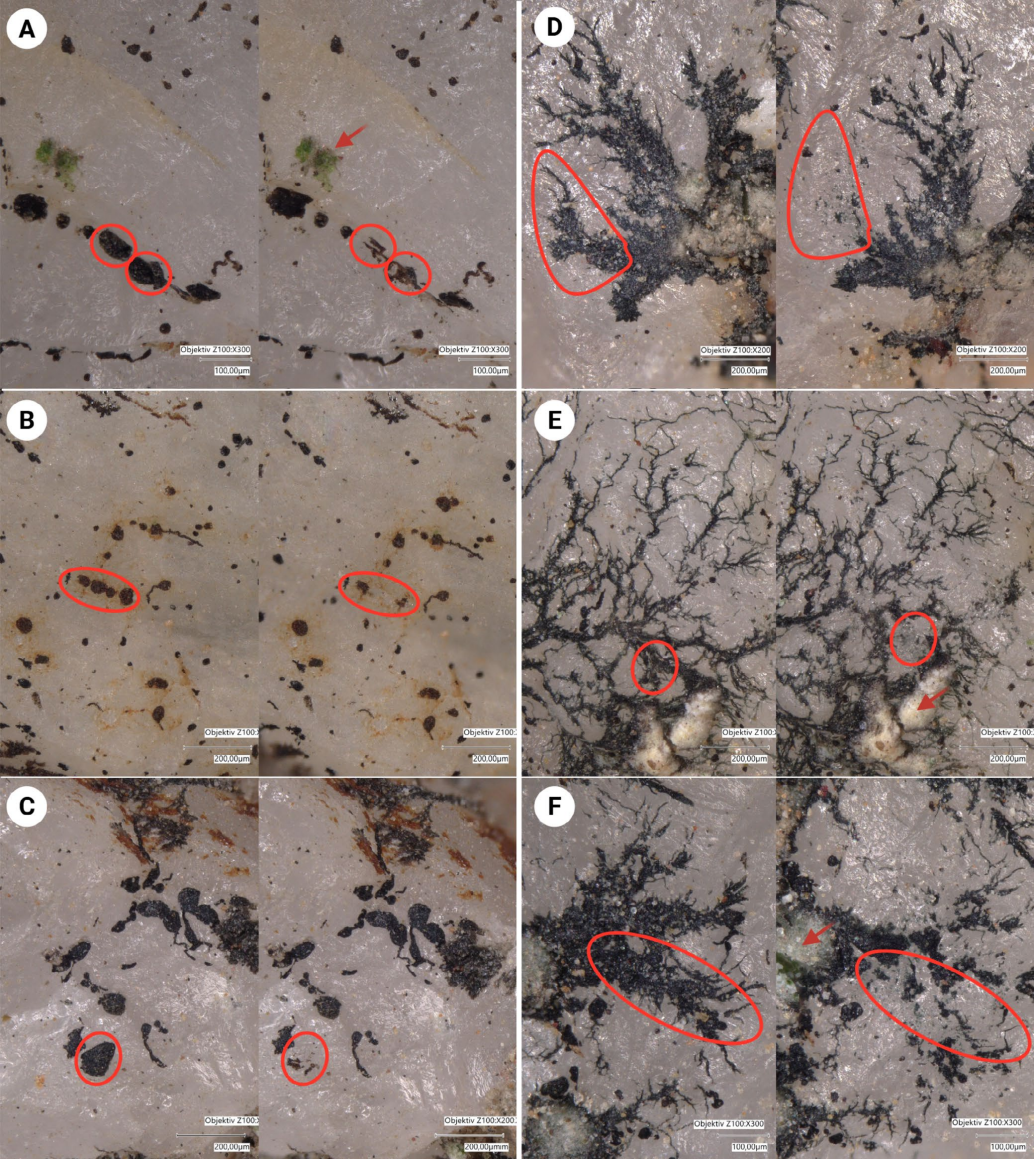
Comparative micro‐manipulative direct PCR approach. (A–F) photo series showing before and after removing biomass with a metal syringe for direct PCR. Arrows indicate photobionts. (A–C) black micro‐colonies and green colony (arrow). (D–F) Prothallus network and mature lichen thallus (arrow).

**FIGURE 7:**
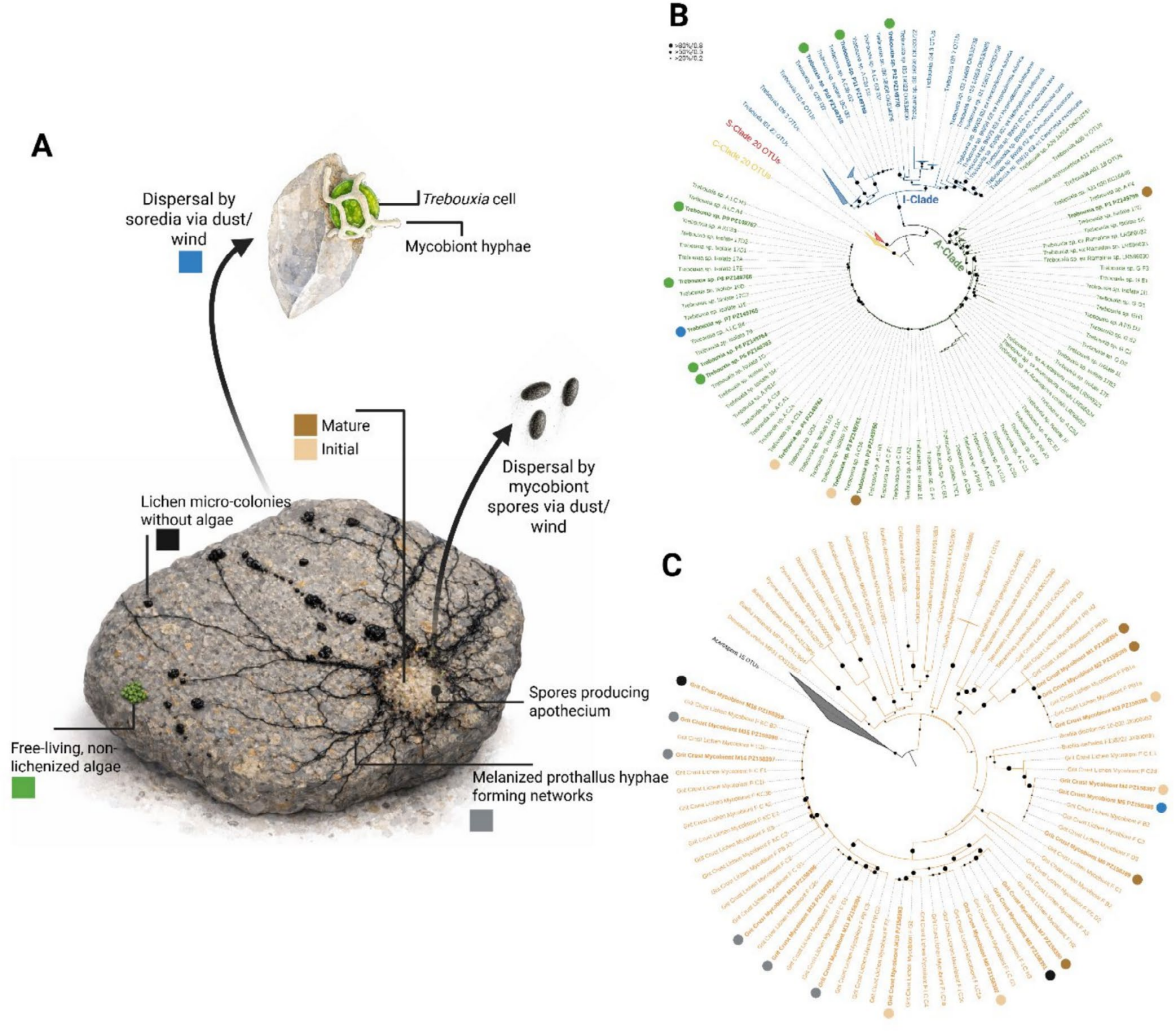
Phylogenetic trees of sequenced lichen structures. (A) Conceptual model of sequential lichen assembly and dispersal in the grit crust. A single colonised grit stone integrates the developmental stages and dispersal pathways inferred in this study. Airborne mycobiont spores (dispersed by wind/dust) germinate on the quartz surface to form melanised, non‐lichenised micro‐colonies and exploratory prothallus hyphal networks. Upon contact with compatible free‐living *Trebouxia* cells, which are present on grit surfaces and within cracks and fissures as environmental photobiont reservoirs, initial thalli form and mature into fully lichenised thalli bearing spore‐producing apothecia. Dispersal operates through multiple vectors: Mycobiont spores and vegetative soredia/thallus fragments (carrying both partners) transported by wind and dust, alongside free‐living algal cells. (B) Phylogenetic tree covering the ITS1, 5.8S, ITS2 gene region of Trebouxia showing the four clades A, I, S and C. Colour codes indicate structures of which the corresponding DNA sequence is derived. Branch support by Bayesian Inference (BI) and Maximum Likelihood bootstrap (ML) is indicated by the size of black circles. Data are reproduced from Jung, Briegel‐Williams, et al. (2024). (C) Mycobiont phylogeny covering the ITS1 gene region derived from direct sequencing of lichen material.

Direct PCR amplification from micro‐sampled structures from grit stones allowed the identification of fungal and algal partners associated with different developmental stages (Table 1; Figures 6 and 7). Mature and initial thalli consistently yielded sequences belonging to lichenised fungi of the family Caliciaceae, as well as sequences of *Trebouxia* photobionts.

**TABLE 1:** Direct PCR results indicating main lichen mycobionts in orange and trebouxioid photobionts in green.

Type	Mycobiont primer ITS1f/LR3	Accession number	Photobiont primer ITS1T/ITS4I	Accession number	Interpretation
Mature thallus	Caliciaceae lichen M1	PZ158384	*Trebouxia* sp. P1	PZ149759	Lichen in full symbiosis
Mature thallus	Caliciaceae lichen M2	PZ158385	*Trebouxia* sp. P2	PZ149760	Lichen in full symbiosis
Initial thallus	Caliciaceae lichen M3	PZ158386	*Trebouxia* sp. P3	PZ149761	Lichen in full symbiosis
Initial thallus	Caliciaceae lichen M4	PZ158387	*Trebouxia* sp. P4	PZ149762	Lichen in full symbiosis
Green colony	—		*Trebouxia* sp. P5	PZ149763	Free‐living *Trebouxia*
Green colony	—		*Trebouxia* sp. P6	PZ149764	Free‐living *Trebouxia*
Dust	Caliciaceae lichen M5	PZ158388	*Trebouxia* sp. P7	PZ149765	Initial lichenisation
Green colony	—	—	*Trebouxia* sp. P8	PZ149766	Free‐living *Trebouxia*
Green colony	—	—	*Trebouxia* sp. P9	PZ149767	Free‐living *Trebouxia*
Green colony	—	—	*Trebouxia* sp. P10	PZ149768	Free‐living *Trebouxia*
Green colony	—	—	*Trebouxia* sp. P11	PZ149769	Free‐living *Trebouxia*
Green colony	—	—	*Trebouxia* sp. P12	PZ149770	Free‐living *Trebouxia*
Mature thallus	Caliciaceae lichen M6	PZ158389	*Trebouxia* sp.	—	Lichen in full symbiosis
Mature thallus	Caliciaceae lichen M7	PZ158390	*Trebouxia* sp.	—	Lichen in full symbiosis
Initial thallus	Caliciaceae lichen	—	*Trebouxia* sp.	—	Lichen in full symbiosis
Initial thallus	Caliciaceae lichen M9	PZ158392	*Trebouxia* sp.	—	Lichen in full symbiosis
Initial thallus	Caliciaceae lichen M10	PZ158393	*Trebouxia* sp.	—	Lichen in full symbiosis
Green colony	—	—	*Trebouxia* sp.	—	Free‐living *Trebouxia*
Green colony	—	—	*Trebouxia* sp.	—	Free‐living *Trebouxia*
Green colony	—	—	*Trebouxia* sp.	—	Lichen in full symbiosis
Green colony	—	—	*Trebouxia* sp.	—	Free‐living *Trebouxia*
Green colony	—	—	*Trebouxia* sp.	—	Lichen in full symbiosis
Green colony	—	—	*Trebouxia* sp.	—	Free‐living *Trebouxia*
Green colony	—	—	*Trebouxia* sp.	—	Free‐living *Trebouxia*
Green colony	—	—	*Klebsormidium* sp.	—	Non‐lichenised green alga
Green colony	—	—	*Desmochloris* sp.	—	Non‐lichenised green alga
Green colony	—	—	*Pseudochlorella* sp.	—	Non‐lichenised green alga
Prothallus	Caliciaceae lichen M11	PZ158394	—	—	Lichen prothallus without algae
Prothallus	Caliciaceae lichen M12	PZ158395	—	—	Lichen prothallus without algae
Prothallus	Caliciaceae lichen M13	PZ158396	—	—	Lichen prothallus without algae
Prothallus	Caliciaceae lichen M14	PZ158397	—	—	Lichen prothallus without algae
Prothallus	Caliciaceae lichen M15	PZ158398	—	—	Lichen prothallus without algae
Black micro‐colony	Caliciaceae lichen M16	PZ158399	—	—	Non‐lichenised phase of lichen mycobiont
Black micro‐colony	Caliciaceae lichen M8	PZ158391	—	—	Non‐lichenised phase of lichen mycobiont
Black micro‐colony	Caliciaceae lichen	—	—	—	Non‐lichenised phase of lichen mycobiont
Black micro‐colony	Caliciaceae lichen	—	—	—	Non‐lichenised phase of lichen mycobiont
Black micro‐colony	Caliciaceae lichen	—	—	—	Non‐lichenised phase of lichen mycobiont
Initial thallus	Caliciaceae lichen	—	*Trebouxia* sp.		Lichen in full symbiosis
Initial thallus	Caliciaceae lichen	—	*Trebouxia* sp.		Lichen in full symbiosis
Initial thallus	Caliciaceae lichen	—	*Trebouxia* sp.		Lichen in full symbiosis
Mature thallus	Caliciaceae lichen	—	*Trebouxia* sp.		Lichen in full symbiosis
Mature thallus	Caliciaceae lichen	—	*Trebouxia* sp.		Lichen in full symbiosis
Mature thallus	*Acarospora conafii*	—	*Trebouxia* sp.		Lichen in full symbiosis
Mature thallus	*Wetmoreana* sp.	—	*Trebouxia* sp.		Lichen in full symbiosis
Mature thallus	*Wetmoreana* sp.	—	*Trebouxia* sp.		Lichen in full symbiosis
Dust	Caliciaceae lichen	—	*Trebouxia* sp.	—	Dispersal of Lichen in full symbiosis by fragments
Dust	Caliciaceae lichen	—	*Trebouxia* sp.	—	Dispersal of Lichen in full symbiosis by fragments
Dust	Caliciaceae lichen	—	*Trebouxia* sp.	—	Dispersal of Lichen in full symbiosis by fragments
Dust	Caliciaceae lichen	—	*Trebouxia* sp.	—	Dispersal of Lichen in full symbiosis by fragments
Dust	Caliciaceae lichen	—	*Trebouxia* sp.	—	Dispersal of Lichen in full symbiosis by fragments

*Note:* Shown are the primer combinations which were used for direct‐PCR from certain structures. If successful, the NCBI accession number is given with the names of the identified organisms. Direct‐PCRs with primers tested and found to be negative are also indicated (—).

In contrast, samples taken from black micro‐colonies and prothallus networks yielded fungal sequences belonging to Caliciaceae but did not contain detectable photobiont sequences.

Green algal colonies sampled independently from the stone surface frequently yielded *Trebouxia* sequences but lacked fungal DNA. Additional algal taxa identified in these samples included *Klebsormidium*, *Desmochloris*, and *Pseudochlorella* (Table 1). Based on literature and previous work, they are not photobionts of lichens of the grit crust environment (Jung, Brand, et al. 2024; Guiry and Guiry 2026).

Dust samples collected from the study area contained both fungal and algal DNA associated with lichen symbionts (Table 1; Figure 7). Several samples yielded sequences of Caliciaceae fungi together with *Trebouxia* photobionts, indicating the presence of lichen fragments containing both partners.

## 4 Discussion

### 4.1 Sequential Lichen Assembly on Grit Stones

The results of this study reveal that the grit crust of the coastal Atacama Desert contains a remarkable diversity of microscopic developmental stages of micro‐lichens occurring simultaneously on individual quartz grit stones (summarised in Table 2). These stages range from isolated fungal micro‐colonies and prothallus networks to early lichen thalli and mature reproductive structures (Figures 3 and 4). Such a spatial continuum of structures supports the hypothesis that lichen colonisation in this system proceeds through a sequential assembly process in which fungal propagules first establish non‐lichenised colonies that subsequently interact with photobiont populations present in the surrounding microhabitat, as commonly suspected for most lichens (Pichler et al. 2023).

**TABLE 2:** Lichen structures and putative functions.

Lichen structure	Description/diagnostic features	Putative function
Mature thallus	Pale crustose to squamulose thallus attached to grit surface; often with clear algal layer and expressed prothallus network	Established symbiotic stage; photosynthesis; sexual and possibly asexual reproduction; stabilisation of colonised grit surface; dust trapping
Apothecia	Black rounded reproductive structures on thalli containing melanised spores	Sexual reproduction; production and release of fungal spores for long‐distance dispersal
Pycnidia	Internal asexual reproductive structures containing non‐melanised spores	Production of asexual propagules; local dispersal and multiplication
Initial thallus	Small pale thalli with visible photobionts; often connected to black micro‐colonies by prothallus hyphae	Early lichenised growth stage; establishment of functional symbiosis after contact between fungus and photobiont
Black micro‐colony	Small rounded melanised structure, usually 20–100 μm, lacking visible algae	Early non‐lichenised fungal stage; likely spore germination and initial establishment on quartz surface
Prothallus network	Venous, melanised hyphal network extending across grit surface; sometimes connected to thalli or micro‐colonies	Exploratory growth; spatial expansion over substrate; bridging between colonisation sites; search for compatible photobionts; anchorage and possible bio‐weathering
Scout hyphae	Fine hyphae radiating from micro‐colonies	Surface exploration; microscale colonisation of new substrate patches; photobiont encounter
Free‐living green colony (*Trebouxia*)	Isolated green colony not visibly associated with fungal hyphae	Environmental photobiont reservoir; potential partner for re‐lichenisation/primary lichenisation
Lichen fragment in air/dust	Fungal and algal DNA co‐detected in airborne dust samples	Symbiotic dispersal unit; transport of already established lichen material by wind/dust
Fungal spore in air/dust	Inferred from apothecia and fungal‐first micro‐colonies	Long‐distance dispersal of the mycobiont; initiation of new colonies on uncolonised grits
Algal cell in air/dust	Inferred from free‐living photobionts and dust transport framework	Dispersal of photobionts; replenishment of environmental algal reservoirs
Cracks/fissures	Fungal hyphae and green algae in deeper areas of the grit lithomatrix	Protected micro‐niche for free‐living algae and lichen structures; photobiont reservoir; site of bio‐weathering

The occurrence of round black fungal micro‐colonies that yielded Caliciaceae sequences but lacked photobiont DNA suggests that these structures represent frequent early germination phases of lichenised fungal spores. Similar early fungal growth stages have been described in experimental studies of lichen development, where germinating spores produce exploratory hyphae before successful contact with a compatible photobiont (Ahmadjian 1993; Honegger 1998). The coverage of micro‐colonies on grit surfaces was similar at two investigated locations, which might be due to comparable climatic factors (shown in Jung et al. 2025) shaping the metabolic activity period.

The venous prothallus networks observed on quartz surfaces may represent such exploratory structures, allowing fungal hyphae to expand across the substrate and increase the probability of encountering suitable algal partners. Once compatible photobionts are contacted, these networks may initiate the formation of initial thalli that later develop into mature lichen structures (Table 2).

The presence of isolated green algal colonies on grit stones, including most belonging to *Trebouxia*, further supports the idea that photobionts occur as free‐living environmental reservoirs (Veselá et al. 2024). Increasing evidence suggests that lichen photobionts are not strictly confined to the lichen symbiosis but can exist independently in soils, rock surfaces and other environmental substrates (Dal Grande et al. 2018; Fernandez‐Mendoza et al. 2020). These environmental populations may play a crucial role in facilitating lichen establishment following fungal spore dispersal. In the grit crust system, the close spatial association between isolated algal colonies and fungal micro‐colonies suggests that such interactions may occur frequently on the surfaces of individual grit stones.

### 4.2 Dispersal Dynamics and Colonisation Processes

Dispersal processes likely play a central role in shaping the spatial organisation and rapid turnover of lichen communities in the grit crust. The detection of both fungal and photobiont DNA in dust samples indicates that wind‐driven dust transport may act as an important dispersal vector for lichen propagules in the Atacama Desert (Azua‐Bustos et al. 2019). Wind transport of microbial propagules is known to be particularly important in arid environments where vegetation cover is sparse and soil surfaces are highly exposed (Pointing and Belnap 2012). In such systems, dust particles can carry fungal spores, algal cells, and fragments of microbial communities across large distances (Flores‐Aqueveque et al. 2010; Azua‐Bustos et al. 2019).

Our results indicate that several dispersal strategies, including various spore types, lichen fragments, or green algal cells, may operate simultaneously in the grit crust system (Table 2). The presence of isolated fungal micro‐colonies supports the dispersal of fungal spores that must subsequently establish symbiosis with environmental photobionts. At the same time, the detection of fungal and photobiont DNA together in dust samples suggests that fragments of mature lichen thalli may also contribute to dispersal.

Such multi‐modal dispersal strategies may be particularly advantageous in hyperarid desert ecosystems where colonisation opportunities are spatially patchy and temporally unpredictable. Wind and dust transport can distribute propagules across the landscape, while the presence of free‐living photobiont reservoirs increases the probability that arriving fungal spores will encounter compatible symbiotic partners. Together, these mechanisms may promote efficient colonisation of newly available substrate surfaces.

### 4.3 Rapid Recovery Potential of Grit Crust Communities

The small size and high abundance of micro‐lichens in the grit crust likely confer a high potential for rapid recovery following disturbance. Desert biocrust communities are often vulnerable to physical disturbance such as trampling, wind erosion, or sediment movement, which can disrupt microbial structures and remove biomass from the soil surface (Liu et al. 2025). However, the grit crust differs from many other biocrust systems in that its dominant lichens occur as extremely small thalli growing on individual grit particles rather than forming continuous crust layers.

This structural organisation may provide several advantages for resilience. First, disturbances affecting individual grit stones are unlikely to remove the entire lichen population from the surrounding area, as adjacent stones may remain intact and serve as sources of propagules for recolonisation. Second, the presence of multiple developmental stages on individual stones indicates that colonisation processes occur continuously across the landscape. Even if mature thalli are removed by disturbance, early developmental stages such as fungal micro‐colonies or prothallus networks may remain and continue to develop into new lichen individuals.

Moreover, the presence of free‐living photobiont populations further enhances the recovery potential of the system. Environmental reservoirs of *Trebouxia* and other green algae, for example in cracks and fissures of the grits (Figure 4D–I), may allow newly dispersed fungal spores to establish symbiosis following disturbance events. In the grit crust, these reservoirs may ensure that suitable symbiotic partners are readily available across the substrate surface.

Together, these factors suggest that the grit crust represents a highly dynamic microbial ecosystem capable of rapid regeneration following disturbance. Rather than forming stable, long‐lived crust structures, the system appears to be characterised by continuous cycles of dispersal, colonisation, and growth occurring at the scale of individual grit particles.

### 4.4 Ecosystem Implications and Productivity Potential

The high density of micro‐lichens observed on grit stones indicates that the grit crust may represent an important contributor to ecosystem productivity in the hyperarid Atacama Desert as previously shown (Jung, Brand, et al. 2024). Although individual thalli are extremely small, their cumulative abundance across the desert pavement surface results in substantial photosynthetic activity with high chlorophyll_a + b_ content exceeding 420 mg^−1^ m^−2^ (Jung, Brand, et al. 2024).

Green algal photobionts such as *Trebouxia* are known to be highly efficient photosynthetic organisms capable of rapidly resuming metabolic activity following hydration events (Sadowsky et al. 2016). In coastal fog deserts, frequent fog inputs provide episodic moisture pulses that allow lichens to activate photosynthesis even in the absence of rainfall (Candotto Carniel et al. 2015). Under such conditions, lichen‐dominated biocrusts can contribute significantly to carbon fixation and nutrient cycling in otherwise biologically sparse landscapes including various ecosystem services such as nutrient acquisition via bio‐weathering activity, which has been documented for the grit crust (Jung, Baumann, Emrich, et al. 2020). The demonstrated dynamics may have important implications for ecosystem functioning in the coastal Atacama Desert. The continuous turnover and regeneration of lichen populations across grit surfaces may sustain a persistent layer of photosynthetically active biomass capable of contributing to carbon cycling and primary productivity in this hyperarid environment. In this context, the grit crust represents not only a unique form of biological soil crust but also a highly adaptable microbial ecosystem that responds rapidly to environmental change.

## 5 Conclusion

Our results reveal distinct developmental stages of micro‐lichens on quartz grit stones of the Atacama Desert grit crust, including melanised fungal micro‐colonies, exploratory prothallus hyphae, isolated algal cells and developing lichen thalli. These observations support a sequential assembly process in which airborne fungal spores colonise mineral particles and subsequently encounter free‐living photobionts, particularly *Trebouxia*, present in the surrounding environment. The combination of multiple dispersal pathways, fungal spores, lichen fragments and algal cells transported via wind and dust likely enables rapid recolonisation of disturbed substrates. Consequently, grit crust communities may possess a high recovery potential following disturbance, contributing to the dynamic landscape coverage and primary productivity characteristic of this extreme desert ecosystem.

## Author Contributions

**Lina Werner:** investigation, writing – review and editing, validation, methodology, formal analysis, data curation. **Patrick Jung:** conceptualization, investigation, funding acquisition, writing – original draft, methodology, validation, visualization, software, formal analysis, project administration, data curation, supervision, resources. **Janina Koziol:** writing – review and editing, software, formal analysis, data curation, methodology.

## Funding

This work was supported by the Deutsche Forschungsgemeinschaft (JU 3228/1‐1, INST 252/27‐1) and the University of Applied Sciences Kaiserslautern.

## Conflicts of Interest

The authors declare no conflicts of interest.

## Data Availability

The data that support the findings of this study are openly available in NCBI Genbank at https://www.ncbi.nlm.nih.gov/.
